# Whistle characterization of long-beaked common dolphin (*Delphinus delphis bairdii*) in La Paz Bay, Gulf of California

**DOI:** 10.7717/peerj.15687

**Published:** 2023-07-19

**Authors:** Óscar Carlón-Beltrán, Lorena Viloria-Gómora, Jorge Urbán R., Sergio Martínez-Aguilar, Simone Antichi

**Affiliations:** Department of Marine and Coastal Sciences, Autonomous University of Baja California Sur, La Paz, Baja California Sur, Mexico

**Keywords:** Bioacoustics, Dolphins, Mexico, Gulf of California, Vocal repertoire

## Abstract

Long-beaked common dolphin (*Delphinus delphis bairdii*) distribution is limited to the Eastern North Pacific Ocean. Its whistle repertoire is poorly investigated, with no studies in the Gulf of California. The aim of the present study is to characterize the whistles of this species and compare their parameters with different populations. Acoustic monitoring was conducted in La Paz Bay, Gulf of California. Recordings were inspected in spectrogram view in Raven Pro, selecting good quality whistles (*n* = 270). In the software Luscinia, contours were manually traced to obtain whistle frequencies and duration. Number of steps, inflection points and contour type were visually determined. We calculated the descriptive statistics of the selected whistle parameters and we compared the results with a dolphins population from the Eastern Pacific Ocean. Permutational multivariate analysis of variance (PERMANOVA) was performed to test the intraspecific variation of the whistle parameters among groups. In the present study the mean values (±*SD*) of the whistle parameters were: maximum frequency = 14.13 ± 3.71 kHz, minimum frequency = 8.44 ± 2.58 kHz and duration = 0.44 ± 0.31 s. Whistles with the upsweep contour were the most common ones (34.44%). The coefficient of variation (*CV*) values for modulation parameters were high (>100%), in accordance with other studies on dolphins. Whistle parameters showed significant differences among groups. Finally, ending and maximum frequencies, duration and inflection points of the whistles recorded in the present study were lower compared with the parameters of the long-beaked common dolphins from the Eastern Pacific Ocean. This study provides the first whistle characterization of long-beaked common dolphin from the Gulf of California and it will help future passive acoustic monitoring applications in the study area.

## Introduction

In the Mexican Pacific, two subspecies of common dolphins are recognized, the short-beaked common dolphin (*Delphinus delphis delphis*) with a cosmopolitan distribution; and the long-beaked common dolphin (*Delphinus delphis bairdii*) with a distribution limited to the Eastern North Pacific (ENP) Ocean (Braulik, Jefferson & Bearzi, 2021; Carretta, Chivers & Perryman, 2011), including the Gulf of California (Urbán et al., 2005). The taxonomy of the common dolphin (*Delphinus* sp.) has been historically troubled. Cunha et al. (2015) noted that because the sympatric/parapatric long-beaked and short-beaked common dolphins off California may not interbreed, the ENP long-beaked common dolphins might be recognized as separate species *D. d. bairdii* (Dall, 1873). However, the molecular analysis of the common dolphins from the ENP (Rosel, Dizon & Heyning, 1994) did not include populations from the contiguous southern regions such as the eastern tropical Pacific and the eastern South Pacific. A complete global review and revision of the common dolphins is still pending and nowadays the Committee of Taxonomy of the Society for Marine Mammalogy (CTSMM, 2023) considers provisionally the long-beaked common dolphins distributed in the ENP as a subspecies *D. delphis bairdii* following the suggestion of Hershkovitz (1966).

Dolphins live in fission–fusion societies and use whistles to communicate during social interactions (Au & Hastings, 2008; Madsen et al., 2012). Whistles can vary in frequency, typically between 1 and 35 kHz (May-Collado & Wartzok, 2008; Richardson et al., 2013). Whistle parameters of dolphins showed intra and interspecific differences and they can be used to classify and distinguish species and populations (Ansmann et al., 2007; Azevedo & Van Sluys, 2005; Morisaka et al., 2005; Rendell et al., 1999). While studies investigating the whistle repertoire of short-beaked common dolphins are relatively abundant (Ansmann et al., 2007; Azzolin et al., 2021; Fearey et al., 2019; Gannier et al., 2020; Gannier et al., 2010; Pagliani et al., 2022; Papale et al., 2014; Petrella et al., 2012), only few focused on the acoustics of long-beaked common dolphins (Caldwell & Caldwell, 1968; Oswald, Barlow & Norris, 2003; Oswald et al., 2007; Oswald et al., 2021a; Oswald et al., 2021b), of which none in the Gulf of California. Indeed, in the study area the investigations on this species have mainly been isotopic analyses and population studies (Aurioles-Gamboa et al., 2013; Niño Torres et al., 2006; Vidal & Gallo-Reynoso, 2012). A study focused on the acoustics of the long-beaked common dolphin will allow for comparisons with other species inhabiting the same area, and will help their acoustic identification using passive acoustic monitoring in the future.

Here we present the first whistle characterization of long-beaked common dolphins from the Gulf of California. The aim of this study is to describe frequencies, duration and modulation parameters of the whistles of long-beaked common dolphins encountered in La Paz Bay and to compare our results with a different population inhabiting the Eastern Pacific Ocean.

## Materials & Methods

**Figure 1:**
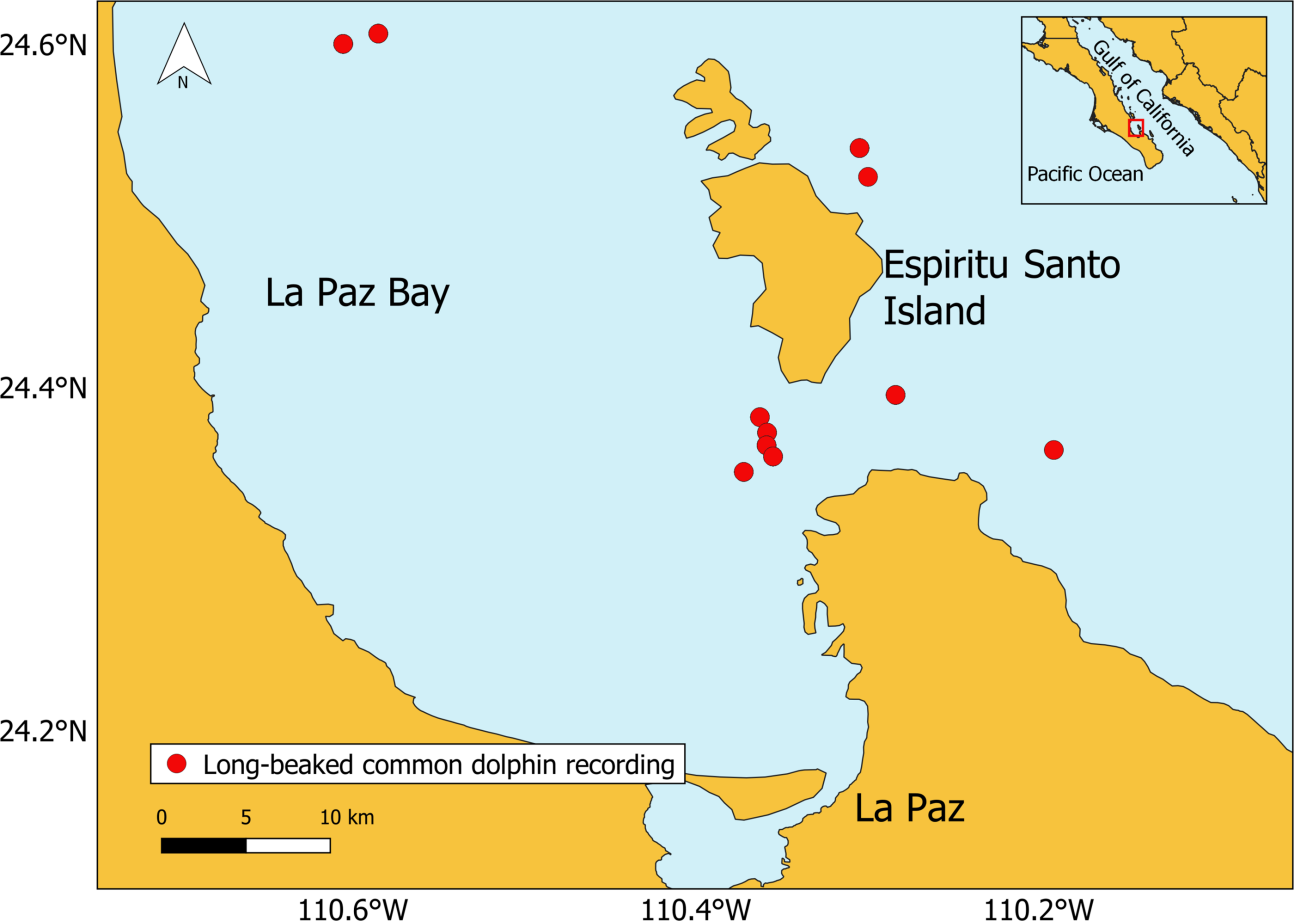
Location of the long-beaked common dolphin recordings (generated with QGIS, version 3.6.3). The base map shapefile of Mexico is provided by Comisión Nacional para el Conocimiento y Uso de la Biodiversidad (CONABIO).

Data were collected in La Paz Bay (Fig. 1), Gulf of California, Mexico, between November 2020 and September 2021. Surveys were conducted in daylight hours during calm sea conditions (Beaufort scale ≤ 2), with a motorized research vessel (7.3 m long, 75 HP). Groups of long-beaked common dolphins were recorded with the engine off, using Reson TC4013.1 omnidirectional hydrophone (sensitivity −211 dB Rms ± 3dB re 1 V/µPa, 1 Hz to 170 kHz) connected to a preamplifier Reson VP2000 Voltage EC6081 (50 dB gain, 500 Hz high-pass filter, 50 kHz low-pass filter). A Marantz PMD661 (data format 24-bits WAV, sampling rate 96 kHz) was used for recording. For each recording session the predominant behavior of the group (displayed by more than 50% of the dolphins) was recorded using continuous scan sampling method (Altmann, 1974; Mann, 1999). The behavior was categorized into five behavioral state categories based on dolphin ethograms (Baker et al., 2017; Bearzi, 1994; Heiler et al., 2016) (Data S1). All behaviors were mutually exclusive. The study was fully observational following the “Guidelines for the treatment of marine mammals in field research” supported by the Society for Marine Mammalogy (Gales et al., 2009). No ethical permit was required by the competent bodies. To ensure consistency of the data collection, the same trained researcher collected the observational data throughout all the study.

**Figure 2:**
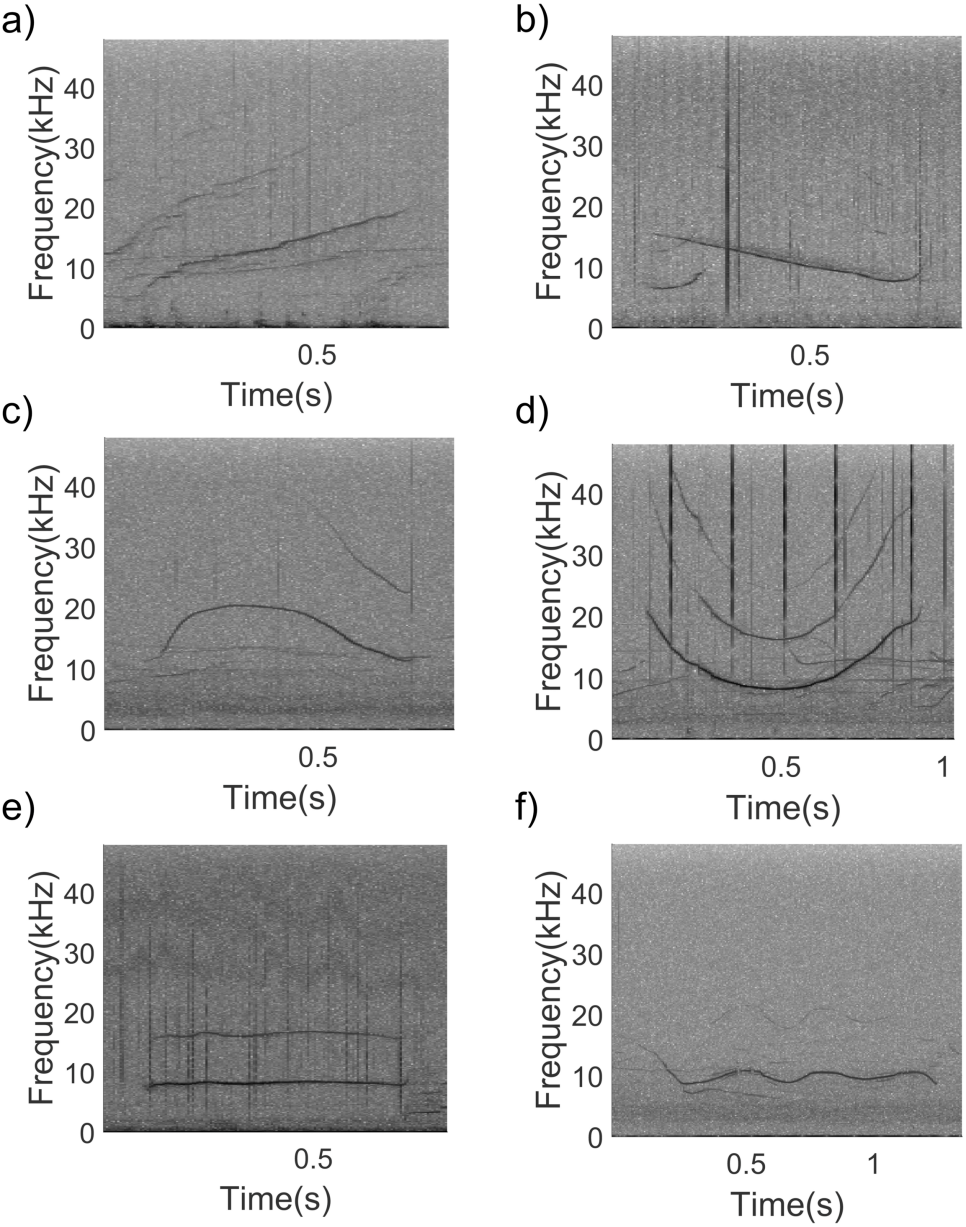
Spectrogram examples of the six whistle contours considered during the study. (A) upsweep, (B) downsweep, (C) convex, (D) concave, (E) constant frequency, (F) sine (1,024 points FFT, Hann window, 50% overlap).

The recording effort was 1 h and 34 min from a total of 5 groups of long-beaked common dolphins recorded (Table 1). No other dolphin species were present during the recordings. A total of 270 good quality whistles (non-overlapped and clearly visible in the spectrogram) were firstly selected in Raven pro (version 1.5 Cornell University, Laboratory of Ornithology, New York) and then whistle frequencies and duration were extracted using Luscinia software (version 2.16.10.29.01) (Lachlan, 2007) as previously described in Antichi et al. (2022b). In addition, number of steps, inflection points, and contour, were visually determined. A step was considered as a period of constant frequency between two periods of the same frequency modulation (*i.e.,* two periods of rising or two periods of falling frequency) (Ansmann et al., 2007; Petrella et al., 2012). Inflection points were defined as shift from falling to rising or rising to falling contour slope (Ansmann et al., 2007; Petrella et al., 2012; Pires et al., 2021). Moreover, each whistle contour was classified into six categories following Ansmann et al. (2007) (Fig. 2).

**Table 1:** Recording effort of the study. Only good quality whistles (non-overlapped with the contour clearly visible in the spectrogram) were considered. To avoid pseudo-replication, whistles with identical time-frequency contours were considered only once.

Dolphin group	Date (dd/mm/yyyy)	No recordings	Recording effort (min)	Group size	No whistles
I	18/11/2020	2	20	150	30
II	09/12/2020	2	16	250	14
III	25/04/2021	1	10	300	10
IV	25/04/2021	1	10	80	3
V	07/09/2021	5	38	140	213

A chi-squared test was used to investigate the relative frequency of occurrence of the six contour forms. Due to the non-normal distribution of the whistle parameters (Shapiro–Wilks, *p* <  0.05) non-parametric tests were used for any further analyses. Permutational multivariate analysis of variance (PERMANOVA) was conducted to test if the whistle parameters varied among dolphin groups. Variables were transformed to y = ln(y+1) to reduce differences in scale among the variables. *P* values for all PERMANOVA tests were calculated based on Euclidean distances using 999 permutations to estimate the probability of group differences. Multilevel pairwise *post hoc* tests (Martinez Arbizu, 2020) with Bonferroni adjustment were performed to calculate differences between pairs of groups. One-sample Wilcoxon test was used to compare the whistle parameters of long-beaked common dolphins collected in our study with the ones recorded in Eastern Pacific Ocean by Oswald et al. (2007). Statistical tests were performed in R software (version 4.2.1; R Core Team, 2022) with RStudio interface (Version 2022.12.0; RStudio Team, 2022).

## Results

**Figure 3:**
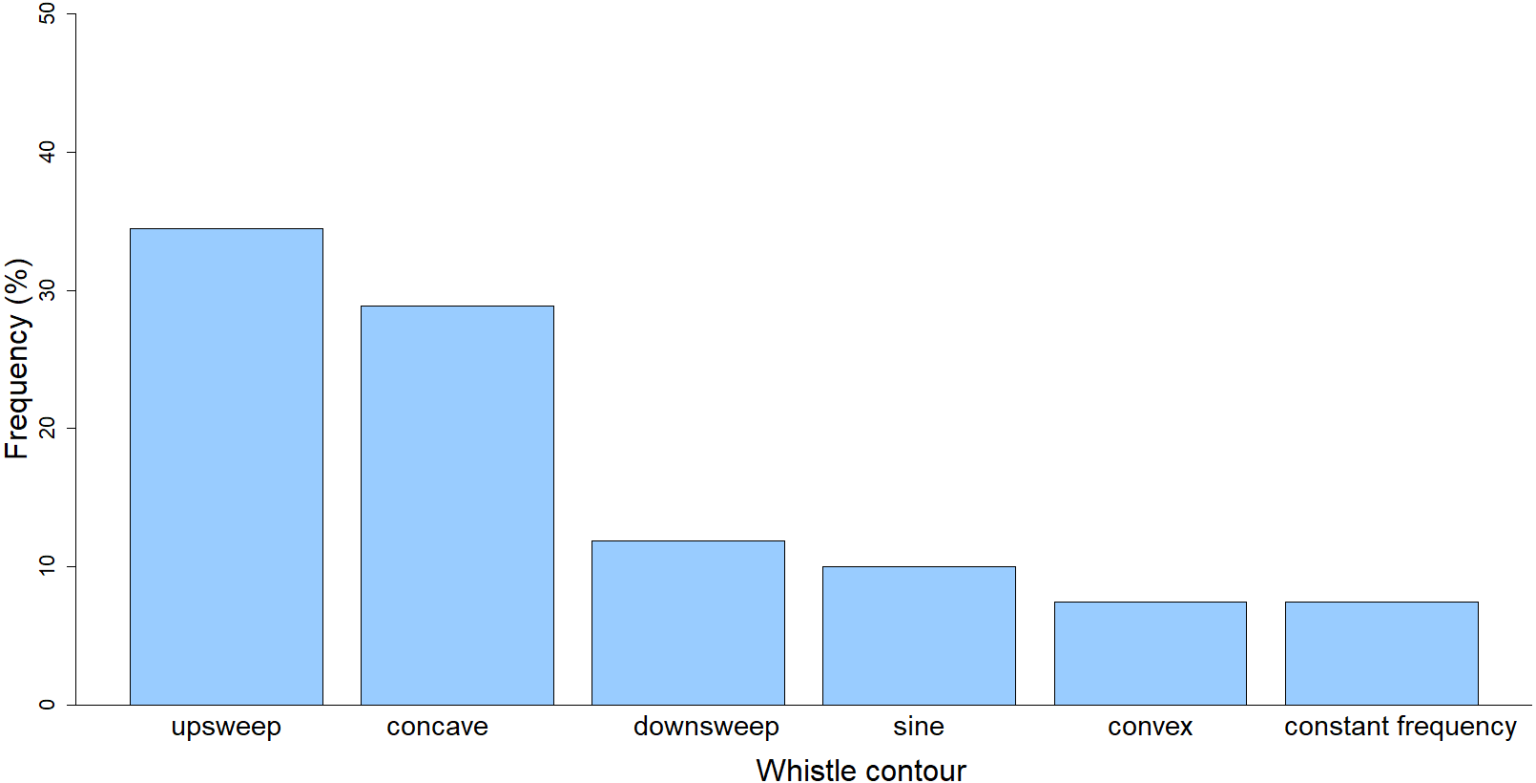
Frequency of whistle contours of the long-beaked common dolphin from the Gulf of California.

Descriptive statistics (maximum, minimum, mean, standard deviation, coefficient of variation) of the whistle parameters were calculated (Table 2). The majority of the whistles (*n* = 260; 96%) were recorded while the dolphins were “traveling” (the group was moving in a consistent direction with regular surfacing intervals) while only during one encounter (*n* = 10; 4%) the predominant behavior was “feeding” (the group was pursuing prey, sometimes with deep dives). Out of the six whistle contour categories, the most common type of contour was upsweep (*n* = 93; 34.44%), followed by concave (*n* = 78; 28.89%), downsweep (*n* = 32; 11.85%), sine (*n* = 27; 10%), convex (*n* = 20; 7.41%), and constant frequency (*n* = 20; 7.41%) (*χ2* = 114.13, *df*. = 5, *p* <  0.001) (Fig. 3).

**Table 2:** Whistle parameters comparison between long-beaked common dolphin from the Gulf of California and from the Eastern Pacific Ocean.

Whistle parameter	Present study (*n* = 270)				Oswald et al. (2007) (*n* = 174)
	Min	Max	Mean ± *SD*	*CV*	Mean ± *SD*
Duration (s)^*^	0.05	2.17	0.44 ± 0.31	70.45%	0.62 ± 0.34
Maximum frequency (kHz)^*^	3.52	26.12	14.13 ± 3.71	26.26%	16.21 ± 4.94
Minimum frequency (kHz)	2.62	18.47	8.44 ± 2.58	30.57%	8.48 ± 2.70
Frequency range (kHz)	0.43	19.93	5.69 ± 3.40	59.75%	–
Starting frequency (kHz)	2.62	26.12	10.81 ± 4.25	39.32%	10.87 ± 4.89
Ending frequency (kHz)^*^	3.28	21.58	12.50 ± 3.78	30.24%	14.46 ± 5.12
Peak frequency (kHz)	3.00	20.55	10.46 ± 2.62	25.05%	–
No. of inflection points^*^	0.00	8.00	0.92 ± 0.99	107.61%	1.59 ± 3.29
No. of steps	0.00	4.00	0.40 ± 0.68	170.00%	–

**Notes.**

* Significantly different parameters between the two long-beaked common dolphin populations (One-sample Wilcoxon test, *p* <0.001).

Whistle parameters showed significant differences among groups (*F* = 4.762, *df* = 4, *p* = 0.001). Five pairs of groups showed significant differences in whistle parameters (Table 3). The group IV showed no differences with the other groups. The whistles recorded in the present study showed lower ending frequency (*V* = 8, 582, *p* <  0.001), maximum frequency (*V* = 7, 786, *p* <0.001), duration (*V* = 7, 142, *p* <  0.001) and inflection points (*V* = 3, 378, *p* <  0.001) than the whistles from Oswald et al. (2007). No differences were found for starting frequency (*V* = 16, 607, *p* = 0.09476) and minimum frequency (*V* = 16, 921, *p* = 0.1429) (Table 2).

**Table 3:** Multilevel *post hoc* pairwise tests with Bonferroni adjustment of the whistle parameters between group pairs.

Group pairs	*R* ^2^	*p*	*padjusted*
I *vs* II	0.023576317	0.402	1.00
I *vs* IV	0.057779319	0.112	1.00
I *vs* III	0.143076262	0.001	0.01^*^
I *vs* V	0.037041288	0.001	0.01^*^
II *vs* IV	0.125803616	0.067	0.67
II *vs* III	0.220604483	0.001	0.01^*^
II *vs* V	0.022609014	0.003	0.03^*^
IV *vs* III	0.132934612	0.153	1.00
IV *vs* V	0.003419664	0.524	1.00
III *vs* V	0.020883659	0.004	0.04^*^

**Notes.**

* Significant differences between group pairs (*p* adjusted <0.05).

## Discussion

This study represents the first whistle characterization of long-beaked common dolphin from the Gulf of California. Indeed, in the study area only the acoustic behavior of the common bottlenose dolphin (*Tursiops truncatus*) was studied (Antichi et al., 2023; Antichi et al., 2022a; Antichi et al., 2022b; Gauger, Caraveo-Patiño & Romero-Vivas, 2021; Gauger et al., 2022). Group size of the groups encountered ranged between 80 and 300 individuals with a mean of 184 dolphins. This is in accordance with the study of Oswald et al. (2021b) that also encountered large groups of long-beaked common dolphins, superior to 100 individuals. The whistles of the recorded long-beaked common dolphin ranged from 2.62 kHz to 26.12 kHz, with durations from 0.05 s to 2.17 s. In the present study, the coefficients of variation (*CV*) of the modulation parameters (inflection points and steps) were greater than 100%. This result indicates high variability in the modulation patterns of the whistles, and might be the consequence of either social or ecological factors or even a combination of both (Azevedo & Van Sluys, 2005; Azzolin et al., 2013; Bazúa-Durán & Au, 2004; May-Collado & Wartzok, 2008; Rendell et al., 1999). The high *CV* values for modulation parameters and low *CV* values for frequencies and duration has been previously reported, not only in *Delphinus* sp. (Azzolin et al., 2021; Panova, Agafonov & Logominova, 2021; Papale et al., 2014) but also in other dolphin species (Azevedo & Van Sluys, 2005; La Manna et al., 2020; Oswald, Barlow & Norris, 2003; Pires et al., 2021; Wang et al., 2013).

In regard to the contours, the upsweep category was the most commonly recorded. This result seems to agree with other studies that found upsweep to be the most common contour in common dolphins, always accounting for around 30% of the whistle composition (Ansmann et al., 2007; Pagliani et al., 2022). However, Petrella et al. (2012) reported downsweep whistles as the most common ones, followed closely by the upsweep contour. The biological meaning of the different contour categories still needs to be fully understood (Bazúa-Durán & Au, 2002) but it seems to be associated with different behaviors (Díaz López, 2011; Hawkins & Gartside, 2010; Kuczaj et al., 2015). Upsweep whistle type was found to be highly associated with social behavior in common bottlenose dolphins (Díaz López, 2011). In Australia, Indo-Pacific bottlenose dolphins (*Tursiops aduncus*) showed high correlation between upsweep whistles and socializing, while sine whistle type appeared to be used for group contact call and was more associated with traveling behaviors (Hawkins & Gartside, 2009; Hawkins & Gartside, 2010). In the present study the majority of the whistles were recorded while the dolphins were traveling. This result might help to disclose the relationship between the traveling behavior and the upsweep contour for this species in the study area. Additional behavioral studies are needed to better associate the different whistle contours to behaviors.

The intraspecific variation of whistle parameters among the recorded groups could be due to the fluid society structure in which this species lives (Tyack, 1986; Bazúa-Durán & Au, 2002; May-Collado & Wartzok, 2008). This variability could represent the ability of dolphins to adapt their whistles to constant changes in their biotic and abiotic environment. It has been previously found that greater variation in whistle repertoire can be expected in dolphins that live in fluid societies (Tyack, 1986; Tyack, 2000). The result could be due to the difference in number of whistles analyzed for each group. A more homogeneous number of whistles per group is needed to better assess the possible intraspecific variation of the whistle parameters.

The whistles recorded in the present study seem to differ from the ones collected by Oswald et al. (2007). Specifically, the whistles of the long-beaked common dolphins from La Paz Bay showed lower ending frequency, maximum frequency, duration and inflection points compared to the ones recorded by Oswald et al. (2007). Whistle variation has been previously reported at interspecific (Oswald et al., 2007; Papale et al., 2021; Steiner, 1981) and intraspecific level, for common dolphins (Ansmann et al., 2007; Azzolin et al., 2021; Gannier et al., 2010; Papale et al., 2014; Petrella et al., 2012) and other dolphin species (Akkaya et al., 2023; Azzolin et al., 2013; Luís et al., 2021; May-Collado & Wartzok, 2008; Yuan et al., 2021). The high plasticity of dolphin whistles can be attributed to many factors, including geographical variability, behavioral state, general environment, group size, social context and individual variability (Bazúa-Durán & Au, 2002; Bazúa-Durán & Au, 2004; Camargo Jr et al., 2006; La Manna et al., 2020; May-Collado & Wartzok, 2008; May-Collado, 2010).

Characterizing the whistle parameters of the long-beaked common dolphin, together with the whistles of the oceanic ecotype of common bottlenose dolphin in the study area (Antichi et al., 2023), will eventually assist the acoustic identification of these two species that share the same oceanic habitat, using passive acoustic monitoring. The whistles of the long-beaked common dolphins from this study and the oceanic common bottlenose dolphins (Antichi et al., 2023) appear to be, after a preliminary analysis, distinguishable from each other based on the lower duration and frequency range of the former. Additional studies would help to effectively differentiate the two species, especially when combining the differences between whistle frequency parameters and whistle contours.

## Conclusions

This study presents the first whistle characterization of long-beaked common dolphins from the Gulf of California, bringing us closer to filling the knowledge gap of this poorly studied species, while also providing important information for future studies about its taxonomic status. Whistles produced by long-beaked common dolphins from the Gulf of California differ from the ones recorded in the Eastern Pacific Ocean. Future comparison of the whistle parameters (duration, frequencies and modulation) and contours between the long-beaked common dolphins and the oceanic ecotype of common bottlenose dolphins in the study area may enable their acoustic identification through passive acoustic monitoring. The identification of this species would be crucial to design more focused conservation actions.

##  Supplemental Information

10.7717/peerj.15687/supp-1 Supplemental Information 1 Dolphin ethogram

10.7717/peerj.15687/supp-2 Supplemental Information 2 Raw dataset

10.7717/peerj.15687/supp-3 Supplemental Information 3 R studio script used for the analysis

## Additional Information and Declarations

### Field Study Permissions

The following information was supplied relating to field study approvals (i.e., approving body and any reference numbers): The research was conducted with the permit issued by the Secretariat of Environment and Natural Resources of Mexico (SEMARNAT) (Permit SGPA/DGVS/00657/21.).

### Data Availability

The following information was supplied regarding data availability: The dolphin ethogram, the raw data and the script used for the analysis are available in the Supplementary Files.
