# Supplementary material for: Whistle characterization of long-beaked common dolphin (Delphinus delphis bairdii) in La Paz Bay, Gulf of California
Source: PeerJ. 2023 Jul 19;11:e15687. doi: 10.7717/peerj.15687 (PMC10362854; doi:10.7717/peerj.15687)
Supplement: Supplemental Information 1 [file peerj-11-15687-s001.docx]

Table SM1 Common dolphin ethogram used in the study.

| **Behavior** | **Description** |
| --- | --- |
| Feeding | The group is pursuing prey and feeding (often confirmed by visual observation of the prey). This behavior could be associated with deep diving. Presence of seabirds in the area could be an indicator of feeding behavior. |
| Traveling | The group is moving in a consistent direction with regular surfacing intervals. |
| Socializing | The individuals of the group are in almost constant physical contact with each other, often displaying surface behaviors (breach, leap etc.) and no steadily directional movement. |
| Milling | No net movement; individuals surfacing facing different directions; pod often changes direction; dive intervals vary. May indicate a transitory phase. |
| Resting | Slow, directed movement (slower than speed of vessel); no splashing; closely grouped or in subgroups; short, relatively constant, synchronous dive intervals. |
